# Preliminary Investigation of the Efficacy and Indications of Proton Beam Therapy for Stage IV Pancreatic Adenocarcinoma

**DOI:** 10.7759/cureus.57771

**Published:** 2024-04-07

**Authors:** Hisashi Yamaguchi, Takahiro Kato, Yuki Narita, Michitaka Honda, Koichi Hamada, Yojiro Ishikawa, Ichiro Seto, Yoshiaki Takagawa, Yasuhiro Kikuchi, Masao Murakami

**Affiliations:** 1 Department of Minimally Invasive Surgical and Medical Oncology, Fukushima Medical University, Fukushima, JPN; 2 School of Health Sciences, Fukushima Medical University, Fukushima, JPN; 3 Department of Radiation Oncology, Southern Tohoku Proton Therapy Center, Koriyama, JPN; 4 Radiology, Tohoku Medical and Pharmaceutical University, Sendai, JPN

**Keywords:** ductal pancreatic adenocarcinoma, pancreas cancer metastases, stage iv disease, proton beam therapy, preliminary investigation, pancreatic adenocarcinoma treatment, oligometastasis

## Abstract

Background: The present study aimed to evaluate proton beam therapy (PBT) for stage IV pancreatic adenocarcinoma and its metastases and define the criteria for eligibility.

Materials and methods: We retrospectively evaluated the patients who had a histopathological diagnosis of pancreatic adenocarcinoma, had progressed to stage IV, and underwent PBT for both the primary and some metastatic lesions between 2017 and 2022. PBT was performed using the passive scattering technique.

Results: Sixteen patients (median age, 72 years; range, 55-85 years) were enrolled. All patients had stage IV pancreatic cancer at the initiation of PBT. The median duration from the date of stage IV diagnosis to the initiation of PBT was 5.8 (range, 0.4-13.5) months. Three patients had been diagnosed as having recurrent stage IV cancer at other institutions before their referral to our hospital because they had local recurrence and distant metastases after the resection of the primary tumor. Chemotherapy was as follows: pre-PBT, 0, 1, 2, and 3 lines in 4, 7, 4, and 1 patients, respectively; concurrent with PBT, 0 and 1 line in 11 and 5 patients, respectively; post-PBT, 0 and 1 line in 5 and 5 patients, respectively; and unknown, 6 patients. The median survival times (MSTs) from the date of stage IV diagnosis for the with or without non-irradiated active metastatic tumor were 11.4 and 20.1 months, respectively. Univariate analysis revealed that the performance status (PS) levels (*p* < 0.01), the carbohydrate antigen (CA) 19-9 tumor marker levels (*p *< 0.01), active tumors not treated with irradiation (*p* = 0.02), and with or without post-PBT chemotherapy (*p* < 0.01) were statistically significant factors. Multivariate analysis revealed that the CA 19-9 tumor marker levels (*p*= 0.04), the number of metastatic lesions (*p* = 0.049), and with or without non-irradiated active metastatic tumors (*p* = 0.02) were significant factors.

Conclusion: PBT is indicated when the number of metastases is limited to ≤ 4 lesions and all tumors can be irradiated within the smallest possible number of irradiation fields that can be performed within the patient’s tolerable time, which is a subjective duration that depends on the patient’s reaction during each session. It may be a viable treatment option for patients with oligometastatic pancreatic cancer.

## Introduction

The reported number of targeted radiotherapies performed for the treatment of locally advanced, unresectable pancreatic cancers has increased. The median survival times (MSTs) in patients with locally advanced unresectable pancreatic cancer underdoing various treatments are reported to be as follows: three-dimensional conformal radiotherapy (3D CRT), 8.2-17.4 months [1-5]; stereotactic body radiotherapy (SBRT), 13.9-17.5 months [6-8]; proton beam therapy (PBT), 18-25.6 months [9-11]; and carbon-ion beam therapy (CIBT), 21.5-25.1 months [12-14]. Systemic chemotherapy is currently the standard treatment for stage IV pancreatic adenocarcinoma, and the MSTs of the various regimens have been reported as follows: gemcitabine, 7 months; nab-paclitaxel-gemcitabine, 8.5 months [15]; and folinic acid-fluorouracil-irinotecan-oxaliplatin (FOLFIRINOX), 11.1 months [16].

The concept of the oligometastatic state, proposed by Hellman and Weichselbaum [17], has been described as an intermediate state between limited primary and polymetastatic cancers. In these types of cases, localized metastasis-directed therapies have the potential to prolong survival or even cure the disease [18], and based on promising clinical evidence, SBRT has become widely used in the targeted treatment of local metastases [19]. In April 2020, public medical insurance in Japan began covering SBRT for oligometastatic diseases that meet certain criteria. A previous study reported that the one and two-year overall survival (OS) rates for patients with oligometastatic pancreatic adenocarcinoma treated with SBRT were 79.9% and 46.7%, respectively, and the median MST was 23 months [20]. Therefore, some patients with oligometastatic disease, including those originating from pancreatic adenocarcinoma, may benefit from locally targeted approaches such as SBRT.

Pancreatic primary tumors are associated with significantly higher morbidity [21]. Invasion of the adjacent duodenum leads to gastrointestinal complications, especially bleeding. Invasion of the adjacent bile duct leads to biliary dysfunction, and invasion of the adjacent celiac plexus commonly leads to severe pain. Those symptoms affect the prognosis and the patient's quality of life [22]. A previous report on the use of SBRT for primary tumor management for 20 metastatic pancreatic cancer patients indicated the 12-month local control rate and OS were 43 and 53%, respectively [23].

However, it is unclear what constitutes the oligometastatic state of pancreatic cancer and under what circumstances radiation therapy is appropriate for local pancreatic tumors and metastatic lesions. The present study, therefore, aimed to evaluate the preliminary outcome of PBT for stage IV pancreatic adenocarcinoma and to define the relevant eligibility criteria.

## Materials and methods

Study design and patient enrollment

The present single-center, retrospective cohort study included patients who underwent PBT for stage IV pancreatic adenocarcinoma at our institute between 2017 and 2022. The inclusion criteria were as follows: histopathological diagnosis of a pancreatic adenocarcinoma as the primary tumor; the presence of at least one distant metastatic tumor; and the existence of an unresectable locally advanced primary tumor or an unresectable local recurrence in the pancreas after resection of the primary pancreatic tumor. Each patient underwent positron emission tomography with 2-deoxy-2-[fluorine-18] fluoro-D-glucose integrated with computed tomography (18F-FDG PET/CT) to determine the state of their metastatic tumors.

Exclusion criteria

Patients who did not have a histological diagnosis of pancreatic adenocarcinoma were excluded.

PBT procedure

The dose and fractionation of PBT for each patient were decided via conferences with radiation oncologists and medical physicists and were dependent on the tumor's location and size. In the 3D treatment planning procedure, abdominal CTs were performed with the patient in the supine position, using 2 mm slices and respiratory gating. The gross tumor volume (GTV) was defined as the volume of the pancreatic tumor before treatment, as determined by contrast-enhanced CT, magnetic resonance imaging (MRI), or 18F-FDG PET/CT. The primary clinical target volume (CTV) was defined as the GTV plus a 5 mm margin in all directions, while the prophylactic CTV was defined as all relevant nodal regions from the vicinity of the 11th thoracic vertebra (T11) to the bottom of the second lumbar vertebra (L2), including the porta hepatis, celiac/superior mesenteric artery, and para-aortic/retroperitoneal lymph nodes. The planning target volume (PTV) was defined as the CTV plus a 5 mm margin in all directions and an additional 2-5 mm margin in the craniocaudal direction, depending on the patient’s respiratory movements. Proton beam irradiation was administered during end-expiration using respiratory gating, which was controlled using a laser range finder to monitor the motion of the patient’s body surface.

The following dose constraints were set: gastrointestinal tract, ≤ 55 gray (relative biological effectiveness) (Gy [RBE]); and spinal cord, ≤ 40 Gy (RBE) in 2 Gy fractions (EQD2). In cases in which the PTV overlapped with an organ at risk (OAR), the OAR was prioritized in the PBT planning.

We used a respiratory monitoring system (AZ-733V, Anzai Medical, Tokyo, Japan) and a treatment planning system (XiO-M, Hitachi, Kashiwa, Japan). The proton beam irradiation was performed using passive scattering techniques (i.e., the wobbler method) with Hitachi’s proton-type particle therapy system (Hitachi, Kashiwa, Japan).

Outcomes

The primary outcomes of the present study were two types of OS rates: one is the time from the initiation of PBT, and the other is the time from the date of stage IV diagnosis. OS was defined as the time from the date of initiation of PBT or the date of stage IV diagnosis to death from any cause, evaluated at 6-, 9-, and 12-month follow-ups.

Statistical analysis

**Figure 1:**
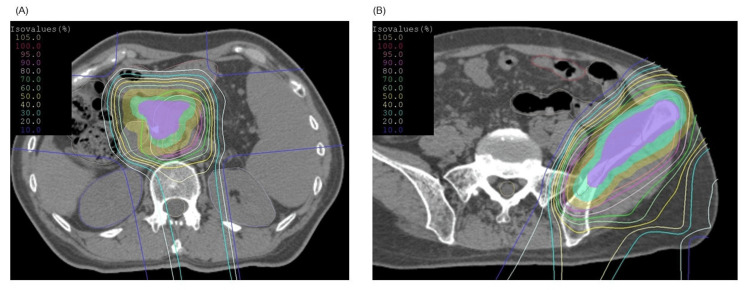
A representative PBT case for a patient with locally advanced pancreatic cancer and bone metastasis. (A) Dose distribution of the PBT using the field-within-a-field technique for patients with locally advanced pancreatic cancer that was adjacent to the GI tract. The large field was irradiated with a total dose of 45 Gy (RBE) at 1.8 Gy (RBE) per fraction. The small field was irradiated with a total dose of 22.5 Gy (RBE) at 0.9 Gy (RBE) per fraction. Therefore, pancreatic cancer was irradiated at a total dose of 67.5 Gy (RBE) at 2.7 Gy (RBE) per fraction. The following dose constraints were set: gastrointestinal tract ≤ 50 Gy (RBE) in EQD2. (B) Dose distribution of the PBT for left ilium metastasis. The target was irradiated with a total dose of 60 Gy (RBE) at 3 Gy (RBE) per fraction.

**Figure 2:**
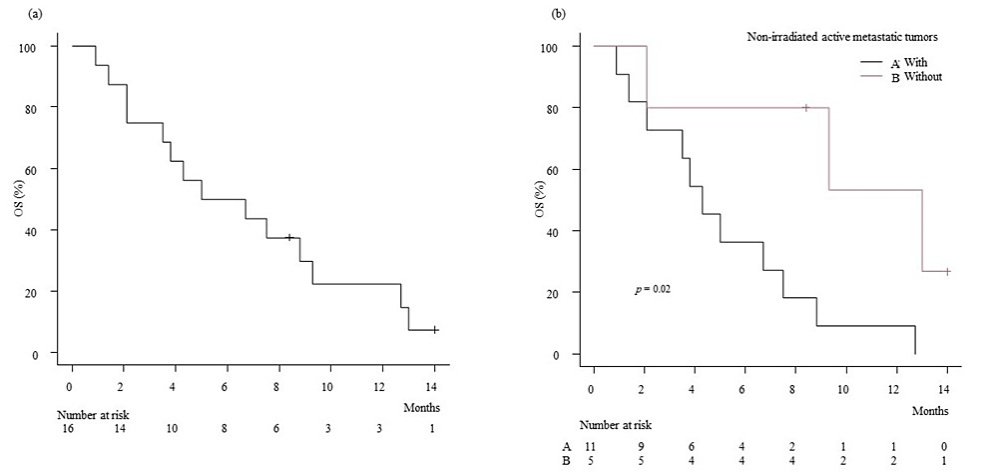
Kaplan-Meier plot of estimated overall survival. (a) from the date of PBT initiation. (b) from the date of PBT initiation for patients with or without non-irradiated active metastatic tumors.

OS rates were calculated using the Kaplan-Meier method. The zero on the X axis in Figure 1 is the start date of PBT, while that on the X axis in Figure 2 is the date of stage IV diagnosis. OS was defined as the length of time from either the initiation of PBT or the date of stage IV diagnosis to the date of death from any cause. The standard therapy for stage IV pancreatic cancer patients is chemotherapy. Therefore, all the patients in the present study first underwent chemotherapy of different durations, followed by PBT. Since the PBT initiation date varies among the patients, OS was calculated for two different periods.

The following factors, which may be related to OS, were investigated: patient’s age; performance status (PS); levels of tumor markers, such as carbohydrate antigen (CA) 19-9 and carcinoembryonic antigen (CEA); GTV of the primary pancreatic tumor (primary GTV); biologically effective dose (BED) of irradiation to the primary tumor; the sum of GTVs of the primary tumor and all metastases (total target GTV); number of metastatic lesions; number of metastatic organs; organ affected by metastasis; with/without peritoneal involvement; with/without ascites; with/without non-irradiated active metastatic tumors; with/without of pre-PBT, concurrent, and post-PBT chemotherapy; and the duration from stage IV diagnosis to initiation of PBT. Because the patients in the present study received various radiation doses in various fractionation schemes, we calculated BED from an α/β ratio of 10 (BED10). 

The cutoff values were estimated using the receiver operating characteristic (ROC) curve and the area under the curve (AUC), while they were calculated with survival ROC curves using present and absent mortality events at six months. The final cutoff value was selected as the point at which the sum of sensitivity and specificity was maximized. Univariate analysis was performed using log-rank tests, and multivariate analysis was performed using the Cox proportional hazards model to determine significant factors. Statistical significance was set at p < 0.05 for all analyses, which were performed using EZR, a graphical user interface for R software (R Foundation for Statistical Computing, Vienna, Austria) [24].

Ethical approval

All of the procedures performed in the present study involving human subjects adhered to the ethical standards of our institutional and national research committees, as well as the 1964 Declaration of Helsinki and its subsequent amendments. We thoroughly explained to candidate patients that PBT is primarily indicated for locally advanced and unresectable pancreatic adenocarcinoma without distant metastasis, and the standard treatment for stage IV pancreatic adenocarcinoma is chemotherapy; therefore, the efficacy of PBT for their cancer remains unknown. We also explained to our candidate patients that PBT would not be covered by either public health insurance because its primary indication is for locally advanced unresectable pancreatic adenocarcinoma without distant metastasis or by advanced treatment coverage of some private insurance. After all, its metastasis treatment indication is for a pancreatic primary tumor that is only stably controlled. After these explanations, only those who agreed to receive PBT at their own expense underwent the therapy. We also placed information regarding the present study, including our contact information, on the website of our proton therapy center as an opt-out option for those who wish to stop participating in this study or wish their data not to be used for this study. We ensured that potential participants knew that no disadvantages would be incurred in the event of refusal to participate, and if no response was received within a certain period, it was considered that informed consent to participate in the study had been obtained. The protocol for the present study was approved by the Ethics Committee of Southern Tohoku General Hospital. The approval number was 566.

## Results

Patient characteristics

A total of 16 patients were enrolled in the present study: 7 men and 9 women, with a median age of 72 (range: 55-85) years. The number of patients with a PS of 0-1, 2, and 3 was 13, 1, and 2, respectively; 11 and 5 patients had Charlson Comorbidity Index (CCI) scores of 0 and 1, respectively. The median CA-19-9 and CEA tumor marker levels were 492 (range, 21-336590) U/mL and 22.4 (range, 3-363) ng/mL, respectively. All patients had stage IV pancreatic cancer at the initiation of PBT. The median duration from the date of stage IV diagnosis to the initiation of PBT was 5.8 months (range, 0.4-13.5). Three patients had been diagnosed as having recurrent stage IV cancer at other institutions before their referral to our hospital because they had local recurrence and distant metastases after the resection of the primary tumor. The patient characteristics are shown in Table 1.

**Table 1:** Patient characteristics. PS: Performance status; CCI: Charlson comorbidity index; CA 19-9: Carbohydrate antigen 19-9; CEA: Carcinoembryonic antigen; PBT: Proton beam radiotherapy. *According to the Eastern Cooperative Oncology Group.

	Number	Range	%
Patients	16		
Male	7		43.8
Female	9		56.3
Age, median	72	(55–85) Years	
PS*			
0–1	13		81.3
2	1		6.3
3	2		12.5
CCI			
0	11		68.8
1	5		31.3
Tumor markers			
CA 19-9, median	492	(21–336590) U/mL	
CEA, median	22.4	(3–363) ng/mL	
Timing of metastases			
Synchronous	16		100.0
Metachronous	0		0.0
Time to PBT from stage IV Diagnosis, median			
	5.8	(0.4–13.5) Months	
Primary treatment			
Chemotherapy	9		56.3
Surgery	3		18.8
Naïve	4		25.0

The median primary GTV was 40.5 (range: 11.9-452.8) mL, and the median total target GTV was 111.6 mL (range: 24.2-798 mL). The number of metastases was as follows: one metastasis (five patients), three metastases (one patient), and five or more metastases (10 patients). The number of organs involved in metastasis was as follows: one organ (eight patients), two organs (seven patients), and three organs (one patient). The organs affected by metastases were as follows: liver (eight patients), lungs (five patients), distant lymph nodes (two patients), bone (three patients), and peritoneum (five patients). Four of the patients developed ascites. In five patients, the primary tumor and all of the metastatic lesions were treated with PBT, while in 11 patients, the primary tumor and some, but not all, of the metastatic lesions were treated with PBT. All of the patients completed the PBT as planned except for one who could not continue due to worsening PS. The median BED10 for the primary site was 85.7 Gy (range, 2.4-93.9), while it was 115.2 Gy (range, 72-115.2) for liver tumors, 115.2 Gy (range, 115.2-115.2) for lung tumors, 96.6 Gy (range, 72.0-96.6) for lymph nodes, and 79.6 Gy (range, 78.0-81.3) for bone metastases. Tumor characteristics are shown in Table 2. The dose distribution of PBT in a representative patient with locally advanced pancreatic cancer and bone metastasis is shown in Figure 1.

**Table 2:** Tumor and irradiation characteristics. GTV: Gross tumor volume; PBT: Proton beam therapy; BED10: Biological effective dose using the linear-quadratic model with α/β = 10 Gy; Gy (RBE)/fr, Gy (relative biological effectiveness)/fraction.

	Number	Range	%
Primary GTV, Median	40.5	(11.9–452.8) mL	
Total target GTV, Median	111.6	(24.2–798.0) mL	
Number of metastatic lesions			
1	5		31.3
2	0		0.0
3	1		6.3
4	0		0.0
5 or more	10		62.5
Number of organs with metastases			
1	12		75.0
2	3		18.8
3	1		6.3
Site of metastasis			
Liver	8		50.0
Lung	5		31.3
Distant lymph node	2		12.5
Bone	3		18.8
Peritoneum	5		31.3
Ascites			
With	4		25.0
Without	12		75.0
Non-irradiated active metastatic tumor			
With	11		68.8
Without	5		31.3
PBT BED_10_, median (range)			
Primary site (16 field)	85.7	(2.4–93.9) Gy	
Liver (11 field)	115.2	(72–115.2) Gy	
Lung (1 field)	115.2	(115.2–115.2) Gy	
lymph node (4 field)	96.6	(72.0–96.6) Gy	
Bone (2 field)	79.6	(78.0–81.3) Gy	
Primary site dose fraction			
2 Gy (RBE)/ 1fr	1	(Interruption)	6.3
46-56 Gy (RBE)/ 23-28fr	5		31.3
67.5 Gy (RBE)/ 25fr	7		43.8
70-77 Gy (RBE)/ 35fr	3		18.8

Pre-PBT chemotherapy was administered as follows: 0 lines (four patients), one line (seven patients), two lines (four patients), and three lines (one patient). The chemotherapy drugs used were as follows: tegafur, gimestat, and otastat potassium combined drug (TS-1) (one patient), gemcitabine hydrochloride (GEM) (four patients), GEM + nabpaclitaxel (eight patients), and FOLFIRINOX or modified FOLFIRINOX (mFOLFIRINOX) (four patients), and immune checkpoint inhibitors (one patient). During PBT, no chemotherapy was administered for 11 patients, while one line of chemotherapy was administered for five patients (GEM for four patients and mFOLFIRINOX for one patient). Post-PBT chemotherapy was administered as follows: 0 lines (five patients), one line (five patients), and unknown (six patients). Post-PBT chemotherapy was administered as follows: TS-1 (three patients), GEM (one patient), and FOLFIRINOX (one patient). The chemotherapy characteristics are shown in Table 3. 

**Table 3:** Chemotherapy characteristics. TS-1: Tegafur, gimestat, and otastat potassium combined drug; GEM: Gemcitabine hydrochloride; mFOLFIRINOX: Modified FOLFIRINOX; ICI: Immune checkpoint inhibitors.

	Number	%
Previous chemotherapy		
Zero	4	25.0
One line	7	43.8
Two lines	4	25.0
Three lines	1	6.3
Chemotherapy before PBT		
TS-1	1	6.3
GEM	4	25.0
GEM+Nabpaclitaxel	8	50.0
FOLFIRINOX/mFOLFIRINOX	4	25.0
ICI	1	6.3
Concurrent chemotherapy		
Zero	11	68.8
One line	5	31.3
Chemotherapy during PBT		
GEM	4	25.0
mFOLFIRINOX	1	6.3
Post-PBT chemotherapy		
Zero	5	31.3
One line	5	31.3
Unknown	6	37.5
Chemotherapy after PBT		
TS-1	3	18.8
GEM	1	6.3
FOLFIRINOX	1	6.3

Survival outcomes

**Figure 3:**
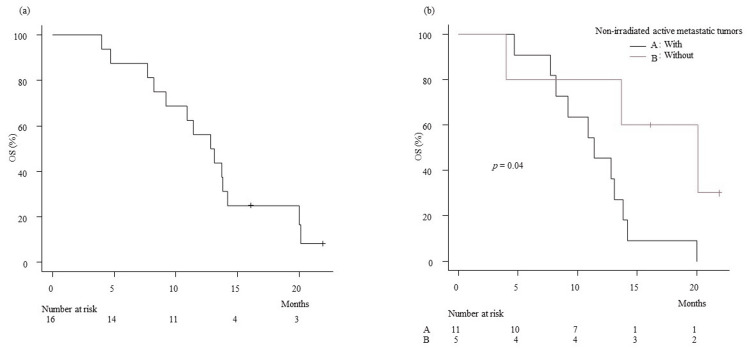
Kaplan-Meier plot of estimated overall survival. (a) from the date of stage IV diagnosis.Kaplan–Meier plot of estimated overall survival. (b) from the date of stage IV diagnosis for patients with or without non-irradiated active metastatic tumors.

The median follow-up period was 5.8 (range, 0.9-14.0) months, and OS rates at 6, 9, and 12 months from the date of PBT initiation were 50.0%, 30.0%, and 22.5%, respectively, with an MST of 5.9 months. The OS rates at 6, 9, and 12 months from the date of stage IV diagnosis were 87.5%, 75.0%, and 56.2%, respectively, with an MST of 13.0 months. The OS rates at six months from the date of PBT initiation in patients with or without non-irradiated active metastatic tumors were 9.1% and 53.3%, respectively (p = 0.02). The MSTs from the date of PBT initiation in patients with or without non-irradiated active metastatic tumors were 4.3 months and 13.0 months, respectively, as shown in Figure 2. The OS rates at six months from the date of stage IV diagnosis in patients with or without non-irradiated active metastatic tumors were 45.5% and 80.0%, respectively (p = 0.04). The MSTs from the date of stage IV diagnosis in patients with or without non-irradiated active metastatic tumors were 11.4 months and 20.1 months, respectively, as shown in Figure 3.

Safety outcomes

One of the patients did not complete planned PBT due to a decline in PS; however, the remaining 15 patients completed PBT without interruption. No adverse reactions ≥ grade 3 were observed. 

OS-related factors

When the Kaplan-Meier curves for the two groups were drawn using cutoff values obtained from the ROC, statistically significant differences in the log-rank test were observed for the PS levels (p < 0.01), CA 19-9 tumor marker levels (p < 0.01), the with or without non-irradiated active metastatic tumors (p = 0.02), and the with or without post-PBT chemotherapy (p < 0.01). The following differences were not statistically significant: patients’ age; CEA tumor marker levels; primary GTV; BED10 of irradiation for the primary pancreatic tumor; total target GTV, including metastatic lesions; number of metastatic lesions; number of organs affected by metastasis; organs displaying metastasis (liver/other); with or without peritoneal dissemination; with or without ascites; with or without pre-PBT/concurrent chemotherapy; and duration from stage IV diagnosis to the initiation of PBT.

Multivariate analyses of the following were performed using Cox proportional hazards models: PS levels; CA 19-9 tumor marker levels; the number of metastatic lesions; the number of organs affected by metastasis; and with or without non-irradiated active metastatic tumors. To adjust for confounding factors, we included all factors in the multivariate analysis for which the oligometastatic status could be measured, such as PS, tumor marker levels, the number of metastatic lesions, and the organs affected by metastasis. We included with or without non-irradiated active metastatic tumors to evaluate treatment factors. Multivariate analysis was not performed on the factors associated with post-PBT chemotherapy because of missing data. The CA 19-9 tumor marker levels (p = 0.04), the number of metastatic lesions (p = 0.049), and with or without non-irradiated active metastatic tumors (p = 0.02) were statistically significant factors. The results are summarized in Table 4.

**Table 4:** Results of statistical analysis. ROC: Receiver operating characteristic; AUC: Area under the curve; MST: Median survival time; CI: Confidence interval; HR: Hazard ratio; PS: Performance status; CA 19-9: Carbohydrate antigen 19-9; CEA: Carcinoembryonic antigen; GTV: Gross tumor volume; BED10: Biological effective dose using the linear-quadratic model with α/β = 10 Gy; PBT: Proton beam radiotherapy. *According to the Eastern Cooperative Oncology Group.

			ROC			Univariate				Multivariate		
Variables	Median	Range	Cut-off Value	AUC		n	MST (months)	95％ CI	P	HR	95％ CI	P
Age	72	55–85	62	0.55	≤ 62	5	4.3	0.9–NA	0.48			
					> 62	11	7.5	2.1–12.7				
PS*	1	0–3	3	0.58	≤ 2	14	7.1	3.5–12.7	< 0.01	3.60	0.86-15.1	0.08
					> 2	2	1.5	0.9–NA				
CA19-9	367	6–336590	14999	0.77	≤ 14999	12	8.1	3.6–12.7	< 0.01	1	1.0-1.0	0.04
					> 14999	4	1.8	0.9–NA				
CEA	22.4	3–363	28.4	0.88	≤ 28.4	9	8.8	1.4–12.7	0.38			
					> 28.4	7	3.8	0.9–5				
Primally GTV	40.5	11.9–452.8	16.1	0.52	≤ 16.1	2	3.9	3.5–NA	0.22			
					> 16.1	14	7.1	2.1–12.7				
Primally BED10	85.7	2.4–93.9	84	0.56	≤ 84	7	4.3	0.9–8.8	0.7			
					> 84	9	7.5	1.4–12.7				
Total GTV	111.6	24.2–798	102.3	0.66	≤ 102.3	8	8.4	1.4–NA	0.2			
					> 102.3	8	4.1	0.9–8.8				
Number of metastatic lesions	5 ≤	1–5 ≤	5	0.61	≤ 4	6	8.4	0.9–NA	0.66	0.45	0.20-0.99	0.049
					> 4	10	4.7	1.4–8.8				
Number of metastatic organs	2	1–3	2	0.65	≤ 1	8	8	0.9–NA	0.61	0.48	0.09-2.54	0.13
					> 1	8	4.4	1.4–8.8				
Site of metastasis	-	-	Liver			7	3.5	0.9–9.3	0.15			
			Other			9	7.5	2.1–13				
Peritoneum dissemination	-	-	With			5	7.5	2.1–NA	0.54			
			Without			11	5	1.4–12.7				
Ascites	-	-	With			4	5.9	2.1–NA	0.71			
			Without			12	5.9	1.4–9.3				
Non-irradiated target Lesion	-	-	With			11	4.3	1.4–7.5	0.02	57.1	1.9-1694	0.02
			Without			5	13	2.1				
Chemotherapy Before PBT	-	-	With			12	4.7	1.4–9.3	0.37			
			Without			4	7.8	3.8–NA				
Chemotherapy Concurrent PBT	-	-	With			5	8.8	4.3–NA	0.53			
			Without			11	3.8	1.4–9.3				
Chemotherapy After PBT	-	-	With			5	8.8	6.7–NA	< 0.01			
			Without			5	2.1	0.9–NA				
Time to PBT from stage IV diagnosis	5.8	0.4–13.5	5.6	0.63	≤ 5.6	8	8.8	2.1–12.7	0.52			
					> 5.6	8	4.3	0.9–NA				

## Discussion

The present study aimed to evaluate the preliminary results of PBT for the treatment of stage IV pancreatic adenocarcinoma and to determine the eligibility criteria for local radiotherapy. One patient was unable to complete PBT due to the decline in PS; however, all of the remaining patients completed PBT as planned. In most patients, PBT can be performed safely without major adverse events. The MST from the date of stage IV diagnosis in the patients without non-irradiated active metastatic tumors was 20.1 months. The number of metastatic lesions with or without non-irradiated active metastatic tumors was found to be a statistically significant factor.

In the Stereotactic Ablative Radiotherapy for the Comprehensive Treatment of Oligometastases (SABR-COMET) phase II randomized trial, 99 patients with controlled primary malignancies plus 1-5 metastatic lesions, all of which were amenable to stereotactic ablative radiotherapy, were randomly categorized in a 1:2 ratio into palliative standard of care (SOC) treatment (arm 1) and SOC plus SABR (arm 2) groups. The five-year OS rate was 17.7% in arm 1 (95% confidence interval [CI], 6-34%) versus 42.3% in arm 2 (95% CI, 28%-56%; stratified log-rank p = 0.006) [25]. The SABR-COMET trial, however, did not include pancreatic cancer. Additionally, we currently have little knowledge of the efficacy of radiotherapy for the treatment of patients with oligometastases in whom the primary cancer is not controlled.

According to Scorsetti et al. [20], 41 pancreatic adenocarcinoma patients who developed metachronous or synchronous metastases were treated with SBRT. Thirty-three patients (80.5%) underwent surgical removal of the primary tumor. The 1- and 2-year OS rates were 79.9% and 46.7%, respectively, with an MST of 23 months. This excellent prognosis was achieved even in five patients (12.2%) who had active lesions that were not treated with SBRT. The 1- and 2-year OS rates for stage IV pancreatic adenocarcinoma treated with SBRT.

In general, radiotherapy for oligometastatic diseases is limited to cases in which all of the lesions can be irradiated within the patient’s tolerable time, which is a subjective duration that depends on the patient’s reaction during each session. The 11 patients (68.8%) had too many metastatic tumors to irradiate within their tolerable time. As the number of target lesions increases, the irradiation time becomes longer and may exceed the patients’ tolerable time for the day, resulting in a prolonged treatment period. However, to date, there has been no evidence that has shown PBT to be superior to chemotherapy for distant metastases of pancreatic cancer. Therefore, we opted to limit the metastatic PBT treatment period up to approximately five weeks and treat as many metastases as possible within this time constraint. This is the reason why some tumors were unirradiated in these 11 patients. We included them as candidates for pancreatic local treatment because localized treatment of the primary tumor may prolong survival, although we found no evidence to support this hypothesis.

Multivariate analysis revealed that the number of metastatic lesions (p = 0.049) and with or without non-irradiated active metastatic tumors (p = 0.02) were statistically significant factors. Despite the small cohort and the weak statistical power, we consider these factors important because of their statistical significance. PBT is known to be safe for the treatment of metastatic liver [26-31] and lung [32] tumors and PBT can safely treat metastases in multiple organs as long as the number of metastases is small. When a patient has ≤ 4 metastases and the primary tumor can be safely irradiated, PBT is considered to be a viable treatment option.

The present study had some limitations. First, it was not a prospective study, and the patients were treated with various irradiation doses. To eliminate this bias, standardized protocols for dose fractionation should be utilized. Second, the sample size of the present study was small, and we found no evidence to support our findings that localized PBT of the primary tumor is effective in prolonging survival even in patients with non-irradiated active metastatic tumors. Further studies with a larger cohort are needed to establish evidence for this, but patients to be included should be limited to those with four or fewer metastases so that all lesions would be irradiated. Third, there was no unified chemotherapeutic regimen utilized in the present study; there were cases with and without post-PBT chemotherapy (p < 0.01). Chemotherapy was attempted in all of the patients after PBT; however, all of the patients who were unable to receive chemotherapy had systemic conditions such as lower PS that precluded chemotherapy, which may have been confounded by their poor prognostic factors. Although chemotherapy is the standard treatment for stage IV pancreatic cancer, future study designs should compare a combined irradiation and chemotherapy group to a chemotherapy-only group by randomly assigning patients with ≤ 4 metastases to each group, ensuring that all of the tumors are irradiated with a uniform chemotherapy regimen. This is because systemic therapy is generally weakened during local therapy, which might result in a poorer prognosis.

## Conclusions

Given the low incidence of adverse events, PBT might be an additional treatment option for patients receiving chemotherapy for stage IV pancreatic adenocarcinoma with ≤ 4 metastatic lesions. Although the present study involved only a small cohort and used a retrospective study design, we hope our findings based on data analysis and discussion on the use of PBT for stage IV pancreatic carcinoma will encourage a phase II study in the future. However, for stage IV pancreatic adenocarcinoma with oligometastases, chemotherapy is still the standard therapy, and the present study did not clearly show the efficacy of PBT in improving prognosis. Therefore, we cannot conclude that PBT is a better option for patients in this stage. Further studies are necessary to clarify whether local treatment, such as PBT, in addition to systemic chemotherapy, improves the prognosis of stage IV pancreatic adenocarcinoma patients.
